# Plant Defense Responses to Insect Herbivores Through Molecular Signaling, Secondary Metabolites, and Associated Epigenetic Regulation

**DOI:** 10.1002/pei3.70035

**Published:** 2025-02-16

**Authors:** Deepak Kumar Mahanta, J. Komal, Ipsita Samal, Tanmaya Kumar Bhoi, P. V. Dinesh Kumar, Swapnalisha Mohapatra, R. Athulya, Prasanta Kumar Majhi, Andrea Mastinu

**Affiliations:** ^1^ Forest Entomology Discipline, Forest Protection Division Indian Council of Forestry Research and Education (ICFRE)‐Forest Research Institute (ICFRE‐FRI) Dehradun Uttarakhand India; ^2^ Basic Seed Multiplication and Training Centre Central Silk Board Kharsawan Jharkhand India; ^3^ Department of Entomology ICAR‐National Research Centre on Litchi Muzaffarpur Bihar India; ^4^ Forest Protection Division ICFRE‐Arid Forest Research Institute (ICFRE‐AFRI) Jodhpur Rajasthan India; ^5^ Research Extension Centre Central Silk Board Hoshangabad Madhya Pradesh India; ^6^ Department of Agriculture and Allied Sciences C. V. Raman Global University Bhubaneswar Odisha India; ^7^ Forest Protection Division ICFRE‐Institute of Wood Science and Technology (ICFRE‐IWST) Bengaluru Karnataka India; ^8^ Regional Research and Technology Transfer Station (RRTTS) Odisha University of Agriculture and Technology (OUAT) Keonjhar Odisha India; ^9^ Division of Pharmacology, Department of Molecular and Translational Medicine University of Brescia Brescia Italy

**Keywords:** epigenetic regulations, herbivore attack, molecular signaling, plant–herbivore interaction, secondary metabolites

## Abstract

Over millions of years of interactions, plants have developed complex defense mechanisms to counteract diverse insect herbivory strategies. These defenses encompass morphological, biochemical, and molecular adaptations that mitigate the impacts of herbivore attacks. Physical barriers, such as spines, trichomes, and cuticle layers, deter herbivores, while biochemical defenses include the production of secondary metabolites and volatile organic compounds (VOCs). The initial step in the plant's defense involves sensing mechanical damage and chemical cues, including herbivore oral secretions and herbivore‐induced VOCs. This triggers changes in plasma membrane potential driven by ion fluxes across plant cell membranes, activating complex signal transduction pathways. Key hormonal mediators, such as jasmonic acid, salicylic acid, and ethylene, orchestrate downstream defense responses, including VOC release and secondary metabolites biosynthesis. This review provides a comprehensive analysis of plant responses to herbivory, emphasizing early and late defense mechanisms, encompassing physical barriers, signal transduction cascades, secondary metabolites synthesis, phytohormone signaling, and epigenetic regulation.

## Graphical abstract

Plants have developed sophisticated defensive mechanisms against insect feeding techniques over millions of years. These defenses include physical barriers like spines and chemical barriers like secondary metabolites and volatile organic compounds (VOCs). The initial response involves sensing physical and chemical stimuli, leading to hormonal activation and various defensive actions, as detailed in contemporary research on plant–herbivore interactions.

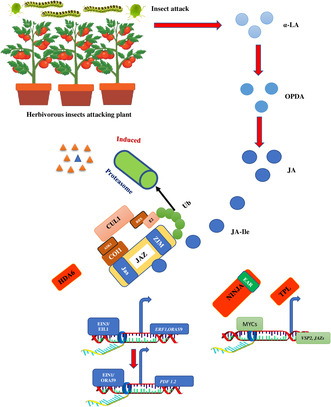

## 1 Background

Approximately one million species are responsible for biotic stress to plants worldwide (Chaudhry et al. 2023; Komal et al. 2023). This ongoing interaction between plants and biotic agents has persisted for over 420 million years, dating back to the Paleozoic era (Labandeira and Wappler 2023). As sessile organisms, plants are unable to move in response to environmental changes, making them highly vulnerable to biotic stressors. Consequently, they have developed various adaptive strategies to mitigate the immediate negative effects of such stressors while maintaining long‐term fitness (Fürstenberg‐Hägg et al. 2013). Over time, coevolution has led to the emergence of diverse biotic agents that are capable of detecting and attacking their host plants using a range of morphological or chemical cues (Mostafa et al. 2022; Bhoi, Samal, Mahanta, et al. 2022; Bhatnagar et al. 2023; Samal et al. 2022, 2023; Al‐Khayri et al. 2023).

Relationships between plants and insects can be categorized into three primary interactions: mutualistic interactions (such as those with pollinating insects), antagonistic interactions (involving herbivores), and interactions with symbiotic organisms like endophytic fungi, including mycorrhiza (Belete 2018; Majhi et al. 2022; Mahanta, Bhoi, et al. 2023; Mahanta, Komal, et al. 2023; Mahanta, Samal, et al. 2023). A substantial body of research has focused on the antagonistic interactions between plants and herbivores to better understand plant defenses. In response to biotic stressors, plants use various defense mechanisms, including both physical and chemical barriers (Hossain et al. 2023; Majhi et al. 2023). Physical barriers, such as the epidermis, thorns, hairs, and trichomes, play vital roles in protecting plants from herbivore damage (Doughari 2015). Upon insect attack, a cascade of chemical reactions is triggered. The initial chemical response in plants involves modifications to the cell wall following insect damage (Kloth et al. 2019). Insect‐emitted signals are detected by specific receptors, which activate immune responses in the host plant (Mostafa et al. 2022; Mahanta et al. 2022).

A critical defense strategy for plant survival is the transmission of long‐distance signals from the site of injury to other parts of the plant. This signaling is essential for activating defense mechanisms that are not only reactive but also anticipatory. Although the terms effector‐triggered immunity (ETI) (Mostafa et al. 2022), pattern‐triggered immunity (PTI) (Sun and Zhang 2021; Singh et al. 2017), and systemic acquired resistance (SAR) have traditionally been associated with bacterial pathogens, they are also applicable in the context of herbivory. Herbivore‐induced signaling pathways often involve similar molecular players and mechanisms, such as jasmonate signaling, which are key to activating plant defenses (Howe and Jander 2008; Spoel and Dong 2012). Research has demonstrated that plants under herbivore attack release volatile organic compounds (VOCs) that attract natural enemies of the herbivores, thereby enhancing resistance to future attacks. This form of immunity is initiated through the recognition of insect oral secretions and signals from damaged plant cells, which activate intricate signaling networks involving calcium ion fluxes and phosphorylation cascades (Howe and Jander 2008).

Calcium ions (Ca^2+^) serve as critical second messengers in plant immune responses, playing a central role in the activation of downstream signaling pathways involved in PTI and ETI (Aldon et al. 2018). In PTI, the recognition of pathogen‐associated molecular patterns (PAMPs) by pattern recognition receptors (PRRs) triggers a rapid influx of Ca^2+^ from extracellular sources, leading to transient calcium spikes. These spikes activate a range of defense‐related genes, induce the production of reactive oxygen species (ROS), and initiate other defense mechanisms. This calcium signature, however, is short‐lived and typically returns to basal levels within minutes (Ashapkin et al. 2020). In contrast, ETI is initiated when plants recognize specific effectors or elicitors from herbivores that suppress host defenses. This recognition leads to sustained elevations of Ca^2+^, which can persist for hours, activating a more robust signaling cascade that culminates in a hypersensitive response (HR) characterized by localized cell death aimed at containing the attacker (Parmagnani and Maffei 2022). Both PTI and ETI involve distinct but overlapping calcium signaling pathways, sharing components like calcium channels and transporters, but differing in their downstream effects due to the nature of the received signals (Howe and Jander 2008). The integration of these signals is essential for the plant's ability to mount an effective defense. Once local defense responses are activated via PTI or ETI, plants can establish SAR, which provides long‐term protection against subsequent attacks. Calcium signaling also plays a pivotal role in SAR, facilitating communication between damaged and undamaged tissues through systemic signals involving salicylic acid (SA) and jasmonic acid (JA) pathways (Costarelli et al. 2020).

Understanding the role of calcium in immune responses is particularly relevant in the context of herbivory, as herbivores often deliver effector proteins through their saliva that can manipulate plant defenses (Zebelo and Maffei 2015). The activation of protein‐phosphorylating mitogen‐activated protein kinases (MAPK) is also implicated in herbivore‐induced plant responses (Cristina Rodriguez et al. 2010; Soares‐Silva et al. 2016). Additionally, ROS and reactive nitrogen species (RNS) generation, as well as NADPH oxidase activation, contribute to the plant's defense against herbivore attacks (Mansoor et al. 2022). Subsequently, phytohormones such as JA, SA, and ethylene are swiftly activated and generated, playing crucial roles in plant signaling pathways that respond to abiotic and biotic stresses (Khokhar et al. 2023; Puri et al. 2023; Kundu et al. 2023). Secondary metabolites (SMs), including VOCs, act as a last line of defense by deterring herbivore feeding and decreasing plant food quality (Mostafa et al. 2022; Belete 2018). The interaction between a plant's genotype and environment also influences insect–plant interactions, and changes in either can lead to different outcomes. Epigenetics plays a significant role in these interactions, as herbivory can induce changes in DNA, histones, and related proteins, influencing plant resistance. Epigenetic modifications, such as DNA methylation and histone modifications, are triggered by biotic stressors and have been widely studied for their impact on plant defense mechanisms (Shah and Joseph 2022). These processes are vital for plant development, maintenance, and resilience, particularly in harsh environments. Prolonged exposure to biotic stress, such as herbivore attack, is a major factor contributing to crop failure and threatens food security (Joseph and Shah 2022; Satapathy et al. 2020). This review highlights the molecular interactions between plants, herbivores, and pathogens, focusing on plant defense signaling and the role of epigenetic control in the early and late stages of plant responses to herbivory.

## 2 An Insight Into Molecular Interaction of Host Plant With Insect Herbivores

Signal transduction pathways in plants rely on several key components, including signaling molecules, receptors, conveyors, transducers, secondary messengers, and transcription factors (TFs). Elicitors, released by herbivores, act as signaling molecules recognized by receptors in host plants. Upon binding to these receptors, the elicitors initiate a signaling cascade that involves signal transducers and secondary messengers, ultimately activating defense‐related genes through TFs. For instance, cabbage plants ( *Brassica oleracea* ) emit specific volatile compounds when infested by cabbage loopers. These volatiles attract parasitoids that prey on the loopers, thereby providing an indirect defense mechanism against herbivory (War et al. 2012). Several TFs play crucial roles in plant defense against herbivores. In *Arabidopsis*, the WRKY TFs regulate defense‐related genes (Kundu and Vadassery 2021). Additionally, MYC and MYB family TFs control the expression of genes involved in antiherbivore responses, including those for terpenoids and flavonoids (Garcia et al. 2021). In tea plants ( *Camellia sinensis* ), the co‐expression of the TFs CsNAC30 and CsTCP11 with CsCHAT1 has been shown to reduce resistance to the herbivore *Ectropis obliqua* (Gu et al. 2024), further illustrating the role of TFs in regulating plant defense mechanisms against herbivory.

The effective detection of microbes and prompt defense responses are essential for plant survival in environments with abundant potentially harmful organisms (Singh, Bhoi, and Vyas 2023; Singh, Bhoi, Khan, et al. 2023). Unlike the human immune system, which relies on specialized defense cells such as lymphocytes, plants rely on each individual cell's ability to recognize pathogens and herbivores. This process is initiated by PRRs, which are plasma membrane proteins capable of identifying molecular patterns unique to pathogens and herbivores. Insects, for example, release herbivore‐associated molecular patterns (HAMPs), which are recognized by plant receptors (Malik et al. 2021). These HAMPs, in conjunction with plant‐derived molecules induced by herbivory, activate a broad spectrum of defensive responses (Cui et al. 2023). These responses can involve various insect‐derived molecules, including saliva, ovipositional fluids, secretions from ventral eversible glands, digestive waste products (frass), and oral secretions (regurgitant). Additionally, plants release endogenous wound signals, such as peptides, in response to insect feeding, which further stimulate defense mechanisms (Kloth and Dicke 2022). Herbivore‐induced cues are highly complex and vary depending on the feeding guild of the insect pest involved. For example, HAMPs and effectors present in insect oral secretions play a significant role in activating plant defenses (Steinbrenner et al. 2022a). Lepidopteran larvae secrete saliva from their labial glands and regurgitant from their mandibular glands to assist with digestion. In contrast, the Coleoptera, which lack salivary glands, can only produce regurgitant secretions (Gedling et al. 2018). These secretions contain various HAMPs and effectors, such as fatty acid conjugates (volicitin), plant‐derived peptides (caeliferins, bruchins, inceptins), and salivary enzymes like β‐glucosidase and glucose oxidase (GOX) (Korada 2017). Although the discovery of HAMPs and effectors continues to grow, identifying the receptors and targets of these molecules within plants remains a challenge (Tables 1 and 2).

**TABLE 1:** Herbivore‐associated molecular patterns (HAMPs).

Plants	Insects	Secretion	HAMP/effector	Response in plants	Reference
*Zea mays*	*Spodoptera exigua*	Regurgitant	Fatty acid‐amino acid conjugates (FACs)	Elicits indirect defense	Prajapati et al. (2020)
	*Manduca sexta*		FACs	Elicits VOCs	Hu et al. (2024)
	*Schistocerca americana*		Caeliferin		Prajapati et al. (2024)
	*Ostrinia nubilalis*	Saliva	Glucose oxidase (GOX)	No effect of GOX on maize	Pingault et al. (2021)
*Solanum lycopersicon*	*Leptinotarsa decemlineata*	Regurgitant	Flagellin (bacteria)	Induction of PR‐1 gene, suppression of JA‐defenses	Satyabrata et al. (2021)
	*Helicoverpa zea*	Saliva	GOX	Elicits direct defense	Parmagnani and Maffei (2022)
	*Ostrinia nubilalis*				Malik et al. (2021)
*Solanum melongena*	*Manduca sexta*	Regurgitant	FACs	Elicits VOCs	Adithya et al. (2024)
*Nicotiana tabacaum*	*Manduca sexta*	Regurgitant	FACs	Elicits VOCs	Malik et al. (2021)
	*Helicoverpa zea*	Saliva	GOX	Suppresses direct defense	
	*Helicoverpa armigera*				
*Arabidopsis thaliana*	*Spodoptera littoralis*	Regurgitant	Porin‐like protein	Membrane channel formation; induction of calmodulin‐like gene	Poretsky et al. (2021)
	*Schistocerca gregaria*		Lipases	Elicits oxylipins, cytosolic calcium levels, mitogen‐activated protein kinases and ethylene	Desaki et al. (2023)
*Brassica oleracea var gemmifera*	*Pieris brassicae*	Regurgitant	β‐Glucosidase	Elicits indirect defense	Hu et al. (2024); Montesinos et al. (2024)
*Vigna unguiculata*	*Spodoptera frugiperda*	Regurgitant	Inceptins	Elicit indirect and direct defenses, JA, SA, ethylene	Steinbrenner et al. (2022b)
*Nicotiana attenuata*	*Manduca sexta*	Regurgitant	FACs	Elicits JA, transcripts and VOCs	Snoeck et al. (2022)

**TABLE 2:** Effects of saliva and glucose oxidase on different host plants.

Plant	Insect	Oral component	Mechanism	Effect	Reference
*Solanum lycopersicon*	*Helicoverpa zea* , *Ostrinia nubilalis*	Glucose oxidase (GOX) from saliva	Amplifies JA signaling by second messenger H_2_O_2_	Induced JA‐regulated defenses and trichomes	Malik et al. (2021)
*Zea mays*	*Ostrinia nubilalis*	GOX	—	No induction of JA‐regulated defenses by GOX alone	Gao et al. (2024)
*Nicotiana tabacum*	*Helicoverpa zea* , *Helicoverpa armigera*, *Helicoverpa assulta*	GOX from saliva	SA‐JA crosstalk	Suppresses nicotine induction and volatile release	Yang et al. (2023)
*Nicotiana attenuata*	*Spodoptera exigua*	GOX	SA‐JA crosstalk	Induced SA, suppressed defenses	Yao et al. (2024)
*Medicago truncatula*	*Spodoptera exigua*	GOX or H_2_O_2_	ETH‐required for SA/NPR1‐mediated	Suppression of trypsin inhibitor and genes that regulate terpenoid biosynthesis	Prajapati et al. (2024)
*Arabidopsis thaliana*	*Spodoptera exigua*	Saliva	Via SA/NPR1, glutathione‐dependent, and DELLA protein	Suppresses JA‐regulated defenses without affecting SA	García‐Marín et al. (2024)
*Arabidopsis thaliana*	*Spodoptera littoralis*	GOX	—	No suppression of ERF/AP2 transcription factor	Yadav and Singh (2024)

Abbreviations: ETH, ethylene; GOX, glucose oxidase; NPR1, non‐expressor of pathogenesis‐related gene 1; SA‐JA, salicylic acid‐jasmonic acid.

Insect oviposition fluids also contribute to plant defense responses. These fluids can attract predators that target insect eggs or trigger the plant to bolster its defenses against subsequent herbivory. For example, oviposition by the sawfly (*Diprion pini*) on Scots pine ( *Pinus sylvestris* ) induces an increase in terpenoid volatile emissions and a decrease in ethylene production (Escobar‐Bravo et al. 2023). In *Arabidopsis thaliana* , oviposition by *Pieris brassicae* induces the expression of defense‐related genes (Aluja et al. 2023). Similarly, the oviposition fluid of *Bruchus pisorum* contains bruchins, which induce tumor‐like growths in *Pisum sativum* , effectively blocking larvae from entering the pod (Santamaria et al. 2018). Furthermore, when *Pieris brassicae* lays eggs on *Brassica oleracea* , it alters the surface chemistry of the leaves, making them more attractive to the egg parasitoid *Trichogramma brassicae* (Afentoulis et al. 2021). This complex interplay between herbivore‐derived cues, plant responses, and defensive signaling pathways is critical for plants to defend themselves effectively against herbivore attack. As research continues, a deeper understanding of these processes will enhance our ability to develop strategies for managing herbivore‐induced plant damage.

## 3 Plant Molecular Defense Mechanisms in Response to Insect Herbivore Attacks

### 3.1 Early Events in the Plant–Insect Interaction

**FIGURE 1:**
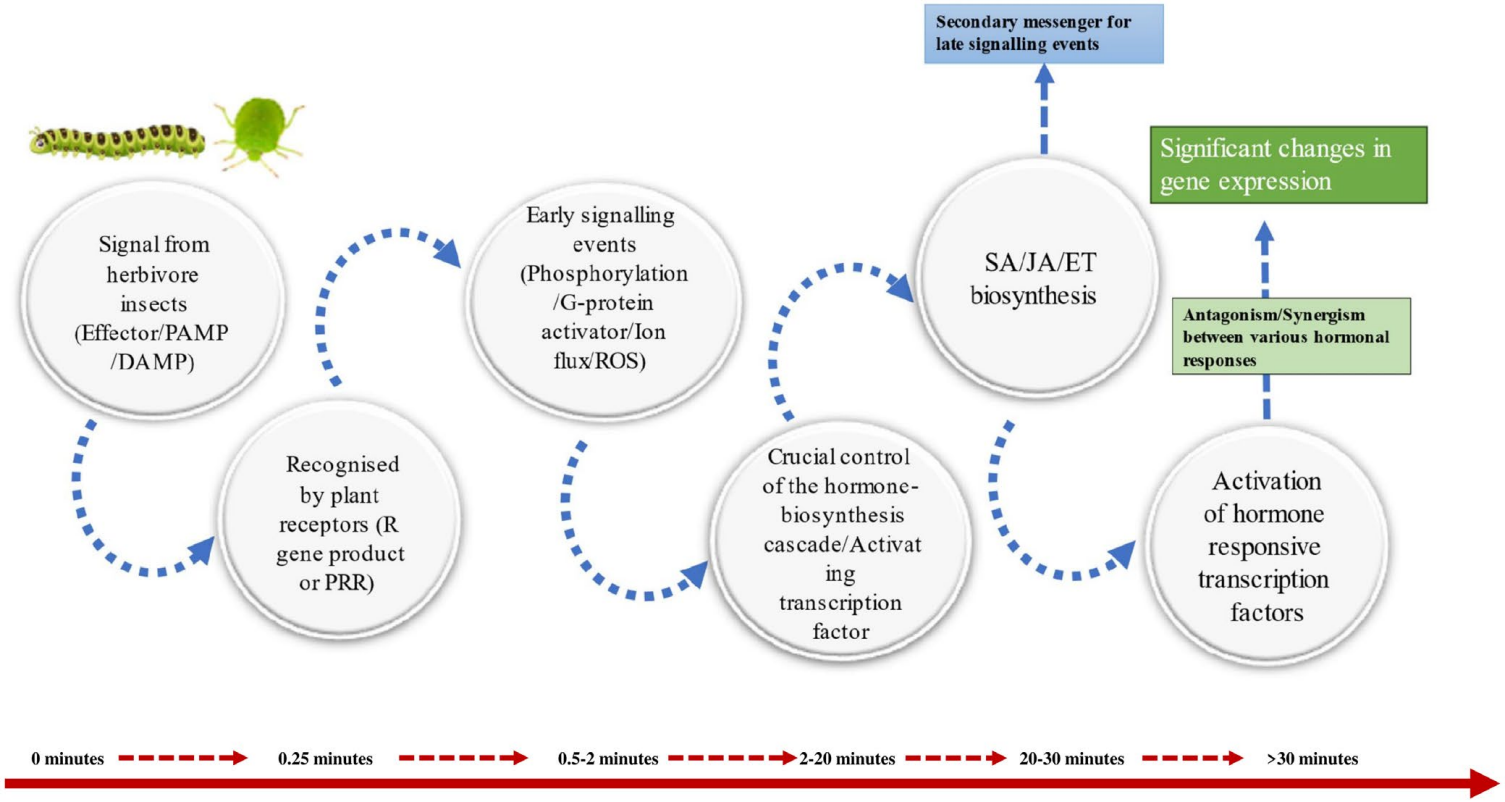
Overview of pathogen/herbivore induced hormonal regulation of genes (adapted from Bigeard et al. 2015).

The majority of research on plant–insect interactions has focused on the proteomics and transcriptomics of the later stages of plant defense (Beran and Petschenka 2022). In contrast, the early events, including signal detection and transduction, remain poorly understood. Immediately following herbivory‐induced damage, changes in the transmembrane potential are observed, which are swiftly followed by alterations in the intracellular calcium concentration (Ca^2+^) and the production of hydrogen peroxide. Within minutes, the activation of kinases and the production of the phytohormone JA can be detected (Figure 1). Gene activation typically occurs about an hour after herbivory, leading to subsequent metabolic shifts (Fürstenberg‐Hägg et al. 2013).

#### 3.1.1 Signal Transduction Involving Change in Membrane Potential and Ca^2+^ Homeostasis

**FIGURE 2:**
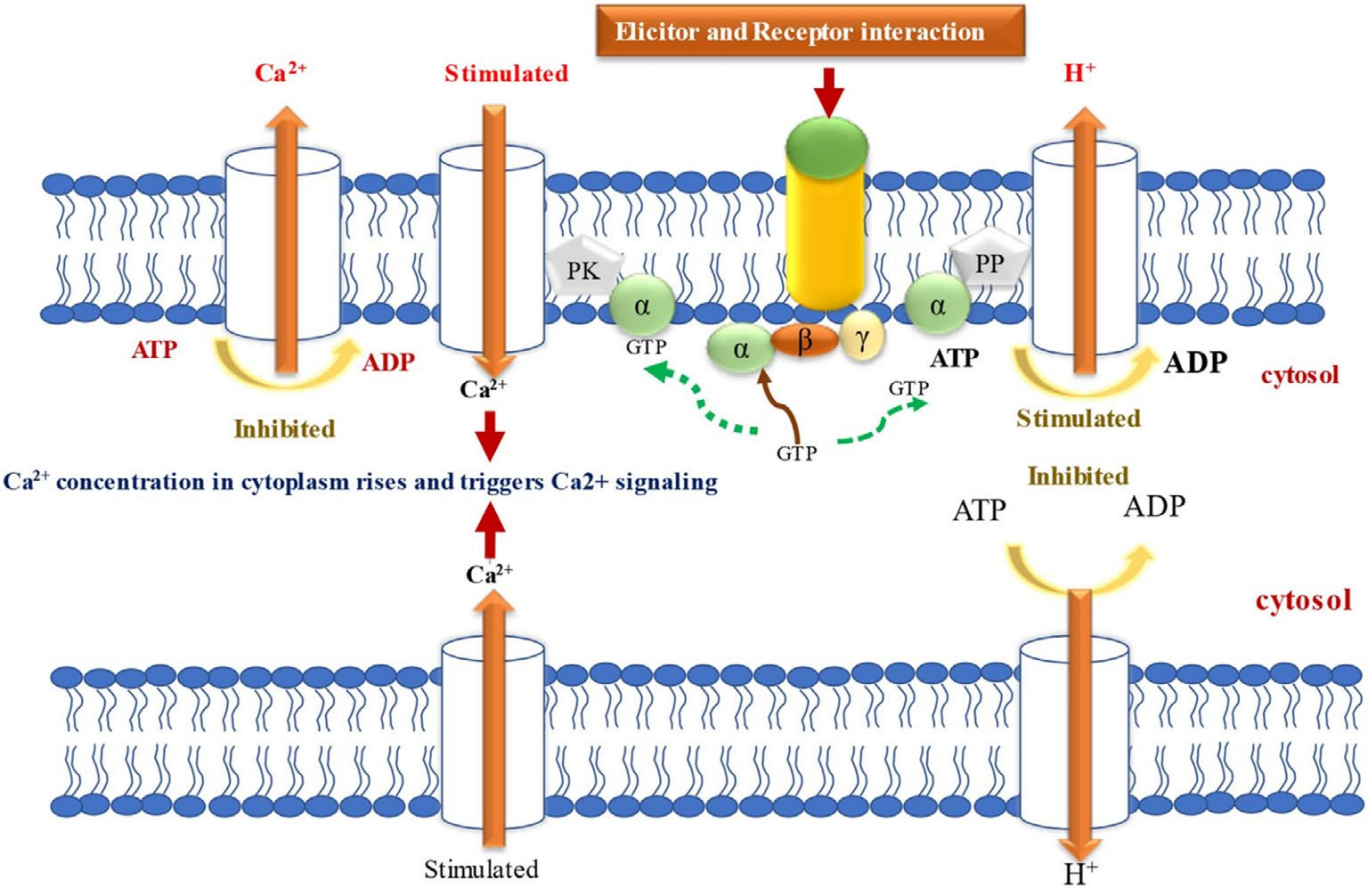
The interaction between elicitors and receptors activates several signaling transduction pathways. In the ion flux transduction pathway, the ligand's interaction to the receptor results in the activation of the G‐protein leads to signal transduction via the activation of phosphatases, resulting in the stimulation of H‐ATPase located in the plasma membrane. This hyperpolarization additionally activates the calcium channels. This G‐protein activates protein kinases that block Ca^2+^ ATPase, resulting in a rise in calcium concentration. Conversely, the inhibition of H^+^ ATPase induces depolarization, subsequently leading to the opening of calcium channels, which ultimately elevates calcium concentration. Dashed lines indicate the alpha component of the active G protein.

Leaf‐feeding insects may trigger the delivery of resistance‐inducing elicitors through their binding to specific plasma membrane receptors (Zebelo and Maffei 2015; Malik et al. 2021). The interaction between resistance elicitors and receptors leads to changes in the electrical potential of the cell membrane, which is regulated by the ion flux across the plasma membrane (Gandhi et al. 2021). These electrical signals propagate from cell to cell, transmitting various messages. Disruptions in ion fluxes result in either depolarization or hyperpolarization of the membrane potential, which in turn activates plant defense mechanisms (Gandhi et al. 2021). For instance, Lima bean ( *Phaseolus lunatus* ) plants damaged by the herbivore Spodoptera littoralis exhibit membrane depolarization (Camoni et al. 2018). Numerous studies have highlighted the role of Ca^2+^ in plants' responses to herbivore attacks. Alterations in membrane potential due to herbivore wounding led to substantial increases in cytosolic Ca^2+^ concentrations (Mostafa et al. 2022). The influx of Ca^2+^ is controlled by protein channels, transporters, and Ca^2+^ sensors in the plasma membrane (Figure 2) (Mostafa et al. 2022). In plants, various calcium‐binding proteins and sensors detect Ca^2+^ signals and regulate subsequent actions, including calcium‐dependent protein kinases (CDPKs/CPKs), calcineurin B‐like proteins (CBLs), CBL‐interacting protein kinases (CIPKs), calmodulins (CaMs)/calmodulin‐like proteins (CMLs), and CDPKs/CPKs (Ghosh et al. 2022).

Calmodulins (CaMs) are calcium‐modulated proteins containing two globular domains, each with two EF‐hand motifs capable of binding a single Ca^2+^ ion. A single CaM molecule can bind up to four Ca^2+^ ions (Noman et al. 2021). CaM‐binding TFs interact with plant hormones to activate defensive responses (Aldon et al. 2018). Through gene interactions, the CaM‐binding TF AtSR1/CAMTA3 negatively regulates SA in infected Arabidopsis plants (Du et al. 2009). Arabidopsis contains 50 CMLs, which are divergent CaMs (Zhu et al. 2015). Overexpression of soybean CMLs (SCaM‐4/‐5) enhances insect resistance (Aldon et al. 2018). CML42 and CML43 are involved in plant resistance to biotic stress (Kanchiswamy and Maffei 2015). For example, the expression of CML42 in Arabidopsis is downregulated by overexpression of JA‐related genes, promoting tolerance to Spodoptera littoralis (Vadassery et al. 2012). CML37 confers protection against *S. littoralis* , indicating its specific role in plant resistance (Scholz et al. 2014). For example, the HAMP inceptin from Spodoptera fugiperda activates the PRR VUINR (Reymond 2021), while the HAMP volicitin from *Spodoptera exigua* attracts parasitoids (Arce et al. 2021). These findings highlight how specific chemical signals from insect herbivores can trigger defensive responses in host plants, enhancing their ability to mitigate herbivory.

In plants treated with lepidopteran OS, insect‐resistant CMLs such as CML9, 11, 12, 16, 17, and 23 are carefully regulated (Vadassery et al. 2012). Calcium/calmodulin‐dependent protein kinases (CCaMKs) were identified in legumes, maize ( *Zea mays* ), and tobacco, but not in Arabidopsis (DeFalco et al. 2009). CCaMKs, unlike CaMs, possess both a CaM‐binding domain and a visinin‐like Ca^2+^‐binding domain with three EF‐hands (Ramachandiran et al. 1997). The CaM‐binding and autoinhibitory domains of CCaMKs overlap. Extracellular ATPases provide energy in stressed plants. The signaling of Ca^2+^, ROS, and nitric oxide (NO) plays a crucial role in extracellular ATP signaling (Tripathi et al. 2018). Ca^2+^‐ATPases facilitate the movement of Ca^2+^ across cell membranes. The P‐type ATPase superfamily, which includes Ca^2+^‐ATPases, is divided into P‐IIB autoinhibited Ca^2+^‐ATPases (ACAs) and ER‐type ACAs, based on their cellular location (ECAs) (García Bossi et al. 2020). In animals, ECAs and ACAs are considered Ca^2+^‐ATPases in the sarcoplasmic reticulum and plasma membrane, respectively (García Bossi et al. 2020). Although ECAs transport Cd^2+^, Mn^2+^, and Zn^2+^, ACAs exclusively transport Ca^2+^ (García Bossi et al. 2020).

#### 3.1.2 Signal Transduction Involving ROS/RNS

**FIGURE 3:**
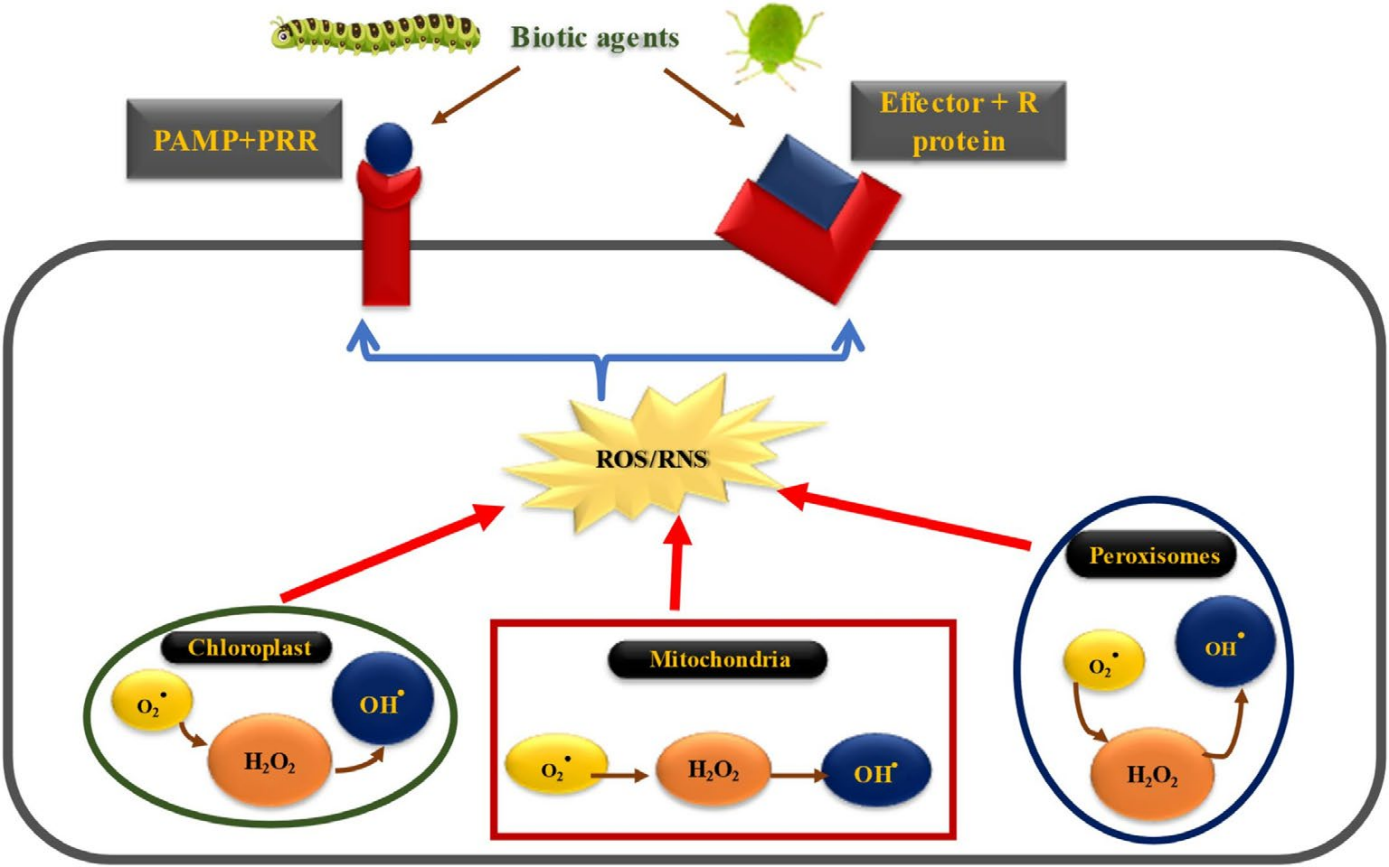
PAMP + PRR (PTI) and Effector +R proteins produce ROS in several organelles like chloroplasts, mitochondria, and peroxisomes. In host‐insect interactions, one of the early signaling events detected in resistant host cells or cells treated with fungal elicitors artificially is the rapid and short‐lived generation of ROS radicals, including superoxide ($O_{2}^{-}$), hydrogen peroxide (H_2_O_2_), and hydroxyl radical (OH). The NADPH‐dependent oxidase enzyme, which is found in plant cell membranes, catalyzes the conversion of O_2_ to $O_{2}^{-}$. Superoxide dismutase enzyme converts oxygen to H_2_O_2_. Eventually, OH radicals are formed through the Fenton reaction.

Salivary proteins influence plant–insect interactions by promoting the accumulation of ROS and triggering cell death. Under biotic stress, increased levels of ROS and intracellular Ca^2+^ ions act as essential signal transducers. Plants generate various types of ROS, including hydrogen peroxide (H_2_O_2_), superoxide anion ($O_{2}^{-}$), hydroxyl radical (OH^−^), peroxynitrite (ONOO^−^), and singlet oxygen (^1^O_2_) (Figure 3). The primary site of ROS production in plants is the plasma membrane, where NADPH oxidase, activated by Ca^2+^ ions, facilitates the formation of $O_{2}^{-}$, which is subsequently converted into H₂O₂ (Poór 2020).

Salivary proteins, such as NlSP1 from the brown planthopper (BPH; *Nilaparvata lugens* ), induce cell death and H₂O₂ accumulation in rice plants, triggering an immune response. These proteins also mediate the interaction between ROS and phytohormone signaling pathways in plants. For example, SA modulates ROS metabolism in mitochondria and promotes ROS generation at high concentrations. SA plays a critical role in SAR, which is essential for plant defense against biotic stress (Poór 2020). Moreover, while SA stimulates ROS accumulation, it also facilitates ROS scavenging to maintain cellular homeostasis (Block et al. 2018).

NO also serves as a key signaling molecule in plant stress tolerance, particularly during herbivore attacks, although its role in RNS signaling remains incompletely understood (Turkan 2018). For instance, during feeding by the pea aphid ( *Acyrthosiphon pisum* ) on *P. sativum* , the plant accumulates NO as part of its defense mechanism (Drzewiecka et al. 2014). Application of exogenous NO to pea plants has been shown to enhance their resistance against aphids, effectively reducing pest populations (Khan et al. 2023). It has been proposed that during abiotic stress, dynamic crosstalk exists between ROS and the NO signaling system, influencing plant stress responses (Molassiotis et al. 2016). However, further research is necessary to elucidate the role of RNS in plant responses to biotic stress and the molecular mechanisms underlying these interactions.

#### 3.1.3 Signal Transduction Involving Kinases

**FIGURE 4:**
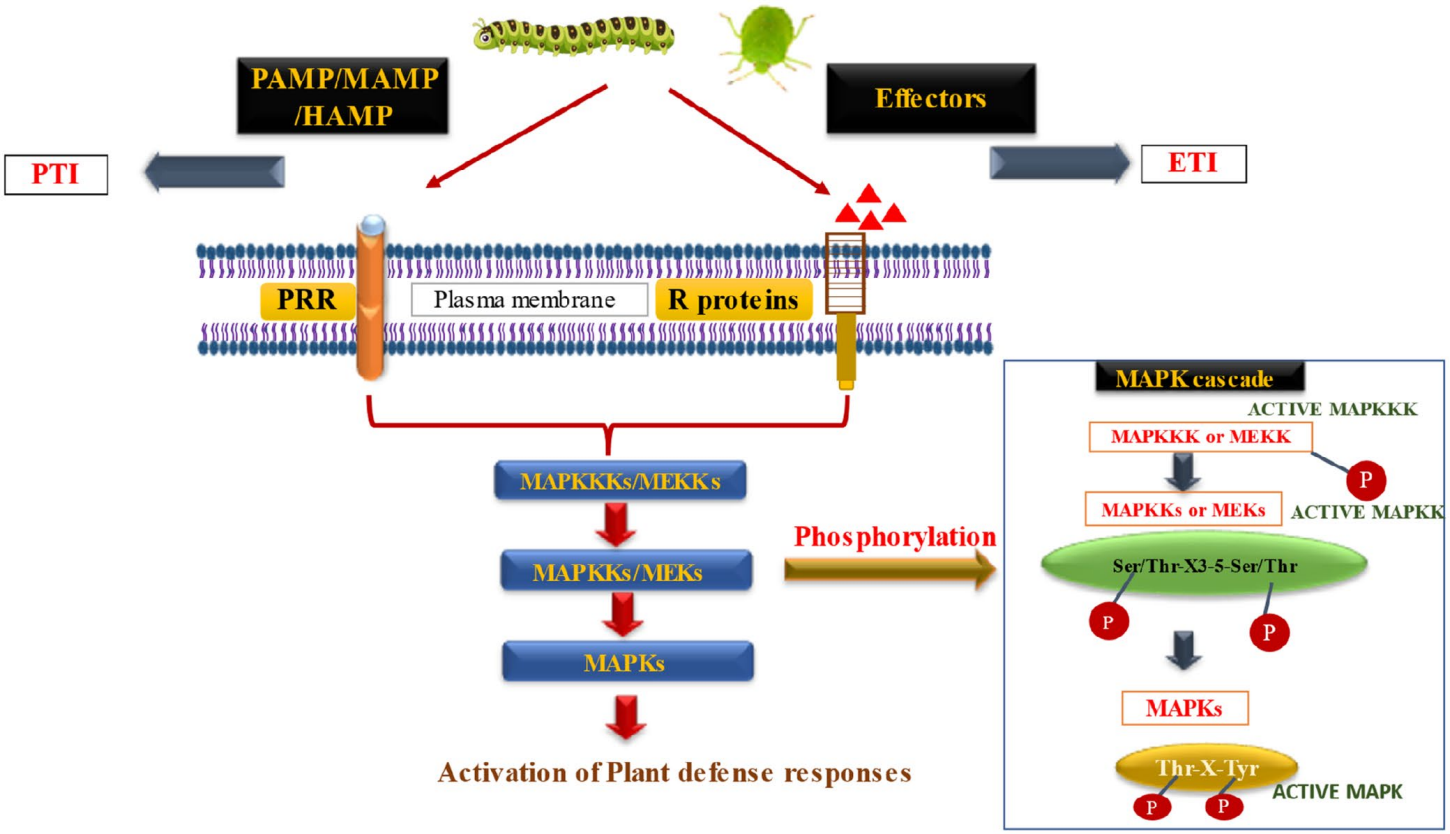
PAMPs recognition by PRR (PTI) and Effectors recognition by R proteins lead to Phosphorylation of MAPK proteins. This PTI and ETI activates MAPKKKs or MEKs through phosphorylation followed by activation of MAPKKs or MEKs through phosphorylation of serine and threonine residues at S/T XXX S/T which further activates MAPKs through phosphorylation at threonine and tyrosine residues at TXY motif between 7th and 8th kinase subdomains. This complete MAPK cascade led to activation of plant defense response.

MAPK cascades are highly conserved signaling pathways in eukaryotes, including plants, that transduce extracellular signals into intracellular responses (Cristina Rodriguez et al. 2010). These MAPK cascades play critical roles in plant defense signaling against biotic stress. Such findings provide valuable insights into the molecular mechanisms driving MAPK activation during plant defense. Plant–pathogen interactions also occur at the level of MAPK cascades. Upon recognizing PAMPs, HAMPs, and pathogen effectors, one of the earliest events in plant defense is the activation of MAPKs (Figure 4) (Heil and Land 2014). MAPK cascades then signal various defensive responses, including stomatal closure, ROS production, activation of defense genes, phytoalexin synthesis, cell wall reinforcement, and the HR, which involves programmed cell death (Ding et al. 2022). However, pathogens utilize effectors to suppress MAPK activity and downstream defense responses, thereby promoting pathogenesis (Zhang et al. 2022).

In eukaryotes, including plants, MAPK cascades are essential for regulating cellular responses to both external and internal signals through sensors and receptors (Zhang and Zhang 2022). Herbivory triggers MAPK activation, and the MAPK pathway is required for the production of ethylene, JA, and the transcriptional regulation of numerous defense genes (Divekar et al. 2022). For instance, silencing the *Lycopersicon esculentum* orthologues of wounding‐induced protein kinase (WIPK) and SIPK reduces JA accumulation in response to herbivory, thereby compromising defense responses against *Manduca sexta* (Tanda and Kaur 2022). Additionally, the tomato homologues of SIPK and WIPK are involved in Mi‐1‐mediated resistance against aphids (Malook et al. 2022). Given the extensive gene family of MAPKs (with 20 MAPKs in the *Arabidopsis* genome), it is anticipated that the number of MAPKs governing plant resistance to various insects will continue to increase (Pitzschke 2015). For example, the tobacco hornworm, *Manduca sexta* , activates SA and WIPKs in both damaged and undamaged leaves when it feeds on *Nicotiana attenuata* (coyote tobacco) (Fürstenberg‐Hägg et al. 2013). WIPKs, in addition to being crucial for JA‐induced responses such as methyl‐JA (MeJA) and ethylene production after wounding, also activate other MAPKs, CDPKs, and TFs. These are essential for the transcription of genes such as ω‐3 fatty acid desaturase (FAD7).

### 3.2 Intracellular/Systemic Late Events in Plant–Insect Interactions

An essential defense mechanism for plant survival is the transmission of long‐distance signals from the site of injury to other parts of the plant. This communication between damaged and healthy tissues is facilitated by systemic signaling (Gandhi et al. 2021). Ion channels play a key role in this process by transferring ions across plasma membranes, enabling long‐distance communication after disruption of the cell membrane and signal transduction pathways. These signals are relayed from the injured areas to neighboring cells, triggering protective responses (Gandhi et al. 2021). For instance, recently discovered non‐selective glutamate receptor‐like channels (GLRs) in *Arabidopsis* are crucial for the transmission of Ca^2+^ signals during herbivory. GLRs in *Solanum lycopersicum* and *Arabidopsis* are both involved in long‐distance signaling (Grenzi et al. 2021). In addition to Ca^2+^ signaling, long‐distance communication is significantly influenced by electrical signals, ROS, and the interaction of ROS with Ca^2+^ (Grenzi et al. 2021). Furthermore, electrical signals generated by mechanical wounding can modulate hormone signaling pathways, including jasmonate synthesis, which is crucial for plant defense against herbivores (Farmer et al. 2020). Specific cell types involved in the propagation of electrical signals following herbivore damage highlight the importance of these signals in activating defense mechanisms (Nguyen et al. 2018). Recent research by Pachú et al. (2023) has examined how plants generate electrical signals in response to mechanical stimuli, playing a critical role in systemic signaling during herbivore attacks (Li, Dou, et al. 2021; Li, Jin, et al. 2021).

**FIGURE 5:**
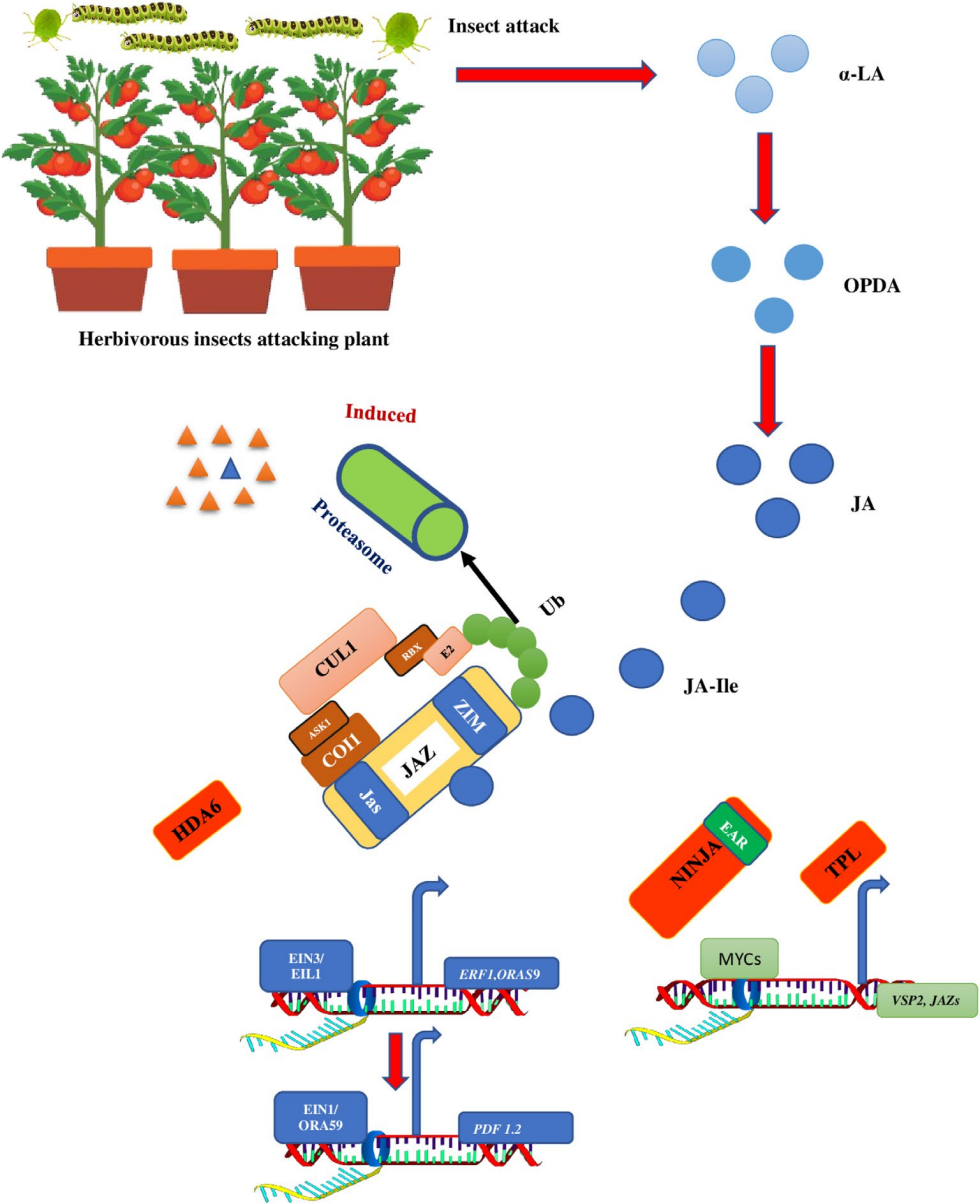
Under insect attack JA is synthesized and converted to JA‐Ile. This JA‐Ile binds with JAZand allow binding of JAZ to COI1. Binding of JA‐Ile to COI1 leads to ubiquitinylation and subsequent degradation of JAZ repressor proteins via the proteasome. In JA stimulated cells, COI1 dependent degradation of JAZ proteins cause disrupts of physical interaction between JAZ proteins and transcriptional activators. This enhances the transcriptional activity of different JA responsive TF and activation of a large number of JA responsive genes.

Previous studies have shown that JA, methyl jasmonate (MeJA), and jasmonoyl L‐isoleucine (JA‐Ile; the bioactive form of JA) can be transported via both phloem and xylem from wounded tissues, accumulating up to several centimeters away in uninjured regions (Zhang, Zhang, and Lin 2020). Additionally, JA is produced in vascular bundles, and when veins are damaged, there is a significant buildup of JA and JA‐Ile (Farmer et al. 2014). The transfer of JA from injured to uninjured areas is demonstrated by the activation of JA in both injured and uninjured tissues after the leaf injury (Schulze et al. 2019). Evidence from *Arabidopsis* indicates that when a shoot is wounded, endogenous JA migrates across phloem tissues, and cis‐12‐oxo‐phytodienoic acid (OPDA), a precursor of JA and its derivatives, is translocated. This triggers the conversion of JA to JA‐Ile and initiates JA signaling in intact roots (Schulze et al. 2019) (Figure 5). Deuterium‐labeled analogues have been used in *tomato* and *tobacco* plants. In both control and injured plants not treated with exogenous JA or JA‐Ile, the exogenous application of JA and JA‐Ile led to significant accumulation of both substances in distal leaves, with JA‐Ile being more mobile than JA (Sato et al. 2011). However, several studies have shown that the induction of JA and JA‐Ile in distant undamaged tissues after plant wounding is due to de novo production, rather than transport from the injury site (Matsuura et al. 2012). In *tomato* plants, accumulated JA and OPDA, as well as the enzymes lipoxygenase (LOX) and allene oxide synthase, which are involved in JA production, are localized in the companion cell‐sieve element complex of vascular bundles, indicating JA biosynthesis in these tissues (Glauser et al. 2008). However, other research has challenged the idea that JA is resynthesized in distant tissues after herbivore attack, arguing that phytohormone distribution is dependent on vascular connections between leaves, since JA concentrations increase in both locally injured and systemically unaffected areas (Zhang, Ménard, et al. 2020; Maithani et al. 2021). There is no systemic induction of the JA precursor OPDA; rather, its concentration rises locally only after prolonged injury (Heyer et al. 2018). Based on these findings, it is unlikely that JA concentrations would rise due to spontaneous biosynthesis (Zhang, Ménard, et al. 2020; Al‐Khayri et al. 2023). To further understand the movement of JA and its derivatives within plant cells and their long‐distance signaling across undamaged regions, further research is needed.

## 4 VOCs Mediated Airborne Defense Signaling

In response to mechanical injury or herbivore feeding, plants release VOCs (Mostafa et al. 2022). Systemic immunity to herbivore attack is induced through long‐distance communication by plants via the emission of VOCs (Scala et al. 2013). VOCs are often produced when cells are disrupted, serving as damage‐associated molecular patterns (DAMPs) and HAMPs that enable plants to detect damage and herbivore attacks (Duran‐Flores and Heil 2016). Following injury, these VOCs are produced de novo, and secondary signals such as oligosaccharides and peptides are subsequently generated (Meents and Mithöfer 2020). For example, when sweet potato plants are injured, the peptide precursor IbHypSys is strongly stimulated, leading to the production of sporamin, a critical defense protein (Chen et al. 2008). In response to DAMPs and HAMPs, *Spodoptera exigua* caterpillar oral secretions significantly increase VOC production in cotton plants (Arce et al. 2021). VOC emission patterns can be categorized into two primary phases: the first occurs shortly after injury (enzymes in leaf tissues), and the second occurs hours later (diverse terpenes and phenolic compounds) (Meents and Mithöfer 2020). Some stored terpenes, including those from pre‐existing secretory structures, are promptly released following tissue injury (Meents and Mithöfer 2020). Other responses to damage include the accumulation of SMs, such as phenolic compounds and tannins, activation of protective oxidative enzymes by MeJA or ethylene, and increased expression and production of proteinase inhibitor genes (Mostafa et al. 2022). Furthermore, the high concentration of VOCs in plants serves as an effective defense mechanism against biotic stress induced by herbivores, acting as repellents. For instance, the highest levels of cereal VOCs repel *Sitophilus granarius* L. and *Tribolium confusum* (Piesik and Wenda‐Piesik 2015; Wenda‐Piesik et al. 2016).

## 5 Plant Defense Against Insect Herbivore Attack by Epigenetic Regulation

Epigenetic regulation plays a crucial role in enabling plants to withstand herbivore attacks and modulate their signaling responses. DNA methylation controls chromatin structure, DNA stability, and gene expression, and is implicated in plant immunity (Kong et al. 2020). For example, when *Pieris brassicae* caterpillars inflict leaf damage on *Brassica rapa* , methylation changes occur in both the leaves and the flowers (Kellenberger et al. 2016). DNA methylation also plays a role in the defense mechanisms of soybean plants against soybean cyst nematodes (Rambani et al. 2020). A recent study found that wheat plants infected with the fungus *Blumeria graminis* f. sp. *tritici* exhibit CHH methylation that regulates their defense mechanisms (Geng et al. 2019). Small RNAs (sRNAs) are involved in plant–herbivore interactions. In plants, short interfering RNAs (siRNAs) are classified into two main types based on their functions: microRNAs (miRNAs), which are derived from single‐stranded stem‐loop precursor structures, and siRNAs, which originate from double‐stranded RNA transcripts (Jeyaraj et al. 2020). Both siRNAs and miRNAs, either individually or in combination, can enhance plant resistance to insects. For instance, in response to attack by the geometrid *Ectropis oblique*, 130 known and 512 novel miRNAs were identified in the tea plant *Camellia sinensis* (Jeyaraj et al. 2017). Similarly, 464 known and 183 new miRNAs were discovered in rice plants after BPH infestations (Wu et al. 2017). In sweet potato, the targets of miR408, such as IbKCS, IbPCL, and IbGAUT, are highly expressed in wounded plants but reduced in transgenic lines overexpressing miR408, demonstrating the role of miRNAs in plant defense (Kuo et al. 2019).

Long non‐coding RNAs (LncRNAs) are involved in several biological and developmental processes, including chromatin remodeling and transcriptional activation (Sun et al. 2018), which enhance plant defenses against biotic stress (Li, Dou, et al. 2021; Li, Jin, et al. 2021). For example, *Nicotiana attenuata* lncRNAs were found to respond to herbivore attack, and their accumulation triggered the release of active jasmonates (Li, Jin, et al. 2021). LncRNAs elicited by armyworm attack have been discovered in treated plants, indicating a direct interaction between herbivores and lncRNAs (Wang, Wu, et al. 2021; Wang, Mostafa, et al. 2021). LncRNAs also mediate plant resistance to aphid damage (Zhang et al. 2019). In plant–pathogen interactions, histone modifications, such as methylation, acetylation, and ubiquitination at the N‐terminal tails, have been observed (Zhi and Chang 2021). Histone acetyltransferases (HATs) and histone deacetylases (HDACs) influence histone acetylation levels. HATs, which transfer the acetyl group from acetyl‐CoA to lysine amino groups, are typically associated with gene activation, whereas HDACs, which remove acetyl groups from histones, are linked to gene repression (Ramirez‐Prado et al. 2018). The complex HAT subunits ELP2 and ELP3 enhance plant defenses due to their acetyltransferase activity (Ramos‐Cruz et al. 2021). Numerous studies have shown that HATs (AtELP2 and AtELP3) and HDACs (AtHDA6, AtHDA9, AtHDA19, AtSRT2, and AtHD2B) contribute to *Arabidopsis* resistance against pathogens (Yang et al. 2020). The acetylation state of cell walls plays a role in plant resistance to phloem‐feeding insects. For instance, pectin acetylesterase 9 (PAE9) enhances *Arabidopsis* defense against phloem‐feeding aphids through DAMP‐induced responses (Kloth et al. 2019). Histone (de)methylation negatively regulates plant immune defense genes (Ramos‐Cruz et al. 2021). Methylation of histones H3K4 and H3K36 promotes the transcription of defense‐related genes, whereas methylation of histones H3K9 and H3K27 represses gene expression (Wang et al. 2019). Histone methyltransferases (AtATX1, AtSDG8, and AtSDG25) and histone demethylases (AtJMJ27 and AtIBM1) play significant roles in regulating plant–herbivore interactions in *Arabidopsis* (Zhang, Ménard, et al. 2020). The process of histone ubiquitination regulates protein interactions with other molecules (Hu et al. 2014). Histone ubiquitination occurs when one or more ubiquitin molecules are attached to the lysine residues of target proteins, mediated by various enzymes (Schnell and Hicke 2003). Ubiquitination exists in two forms: mono‐ubiquitination, which serves as an endogenous signal, and polyubiquitination, which targets proteins for degradation by the 26S proteasome, an ATP‐dependent, multi‐subunit protease complex (Zhang et al. 2015).

## 6 Plant Defense Against Insect Herbivore Attack by Producing SMs

Chemical alterations occur in host plants during plant–insect interactions, such as increased synthesis of SMs, which are essential for regulating plant resistance to herbivores. The metabolite profile of infected plants is altered due to variations in SM concentrations among different compounds (Cai et al. 2014). Plant defense is regulated by five main types of SMs: benzoxazinoids, glucosinolates, terpenes, aromatics, and green‐leaf volatiles (Erb and Kliebenstein 2020). For example, in response to bark beetle attacks, conifers produce accumulated terpenes (monoterpenes) (Khare et al. 2020). The response of herbivores to emitted VOCs varies considerably (Al‐Khayri et al. 2023). Herbivores may be attracted to volatiles released in low to moderate quantities during ecological interactions, which act as ecological signals (Bhoi, Samal, Majhi, et al. 2022; Sujatha et al. 2022). However, additional volatiles released by severely infected plants may deter herbivores (Scott et al. 2020). After plant infection, phenolic compounds, such as coumarins, lignin, flavonoids, furanocoumarins, and tannins, are abundantly produced and contribute to plant defense mechanisms (Gantner et al. 2019). Meta‐analyses have shown that increased levels of phenolic compounds are found in plants infected by beneficial insects, bacteria, and other pathogens (Wallis and Galarneau 2020). Lignin, which enhances tolerance to both biotic and abiotic stress, serves as a physical barrier against herbivory, toughening plant tissues and making them indigestible to insects and other herbivores (Jan et al. 2021; Prabhulinga et al. 2022; Ahmad et al. 2023). The roots of plants produce coumarins, which are primarily responsible for iron absorption. Coumarins also play a role in plant defense against herbivores and pathogens (Stringlis et al. 2019). For example, coumarins from the Apiaceae and Fabaceae families, as well as species like *Cinnamomum cassia* and *Dipteryx odorata* , help prevent herbivory by insects such as *Myzus persicae* , *Spodoptera littoralis*, and *Rhopalosiphum padi* (Barrero et al. 2013). Toxic substances known as furanocoumarins are secreted by certain plant species as a defense against herbivory. These furanocoumarins are most commonly found in Apiaceae and Rutaceae species (Jan et al. 2021). For instance, several *hogweed* plant species (Apiaceae) produce large amounts of furanocoumarins, which protect plants from insect feeding (Harvey et al. 2020). Flavonoids, important plant‐produced SMs, accumulate significantly after herbivore attacks (Shen et al. 2022). Attacks on tea leaves ( *Camellia sinensis* ) trigger the activation of genes associated with flavonoids, causing their accumulation and initiating a defense response against the tea green leafhopper (Zhao et al. 2020). Following herbivore attacks, tannins are also produced to reduce the nutritional value of plant tissues for insects (Perkovich and Ward 2022). In plant–insect interactions, tannins form compounds that reduce nitrogen content, inhibiting insect digestive enzymes and preventing protein hydrolysis (Kariñho‐Betancourt 2018). Conversely, certain insects possess salivary proteins that can bind tannins, reducing their detrimental effects (Perkovich and Ward 2022). Lectins, proteins present in most plants, enhance plant defense during insect encounters by releasing cytokines and other effectors. However, the exact mechanisms of lectin production in response to insect or pathogen attacks remain unclear (Chen et al. 2021).

Sulfur‐containing compounds, such as glucosinolates, glutathione, phytoalexins, and defensins, are crucial for plant defense. Glutathione plays a role in various detoxifying processes in plants, in addition to its signaling function in plant–herbivore interactions (Künstler et al. 2020). For example, in soybean plants infected by the nematode *Heterodera glycines* , glutathione mediates the production of H₂O₂. Low glutathione concentrations elevate H₂O₂ levels, which reduces nematode accumulation (Chen et al. 2020). Glucosinolates are found in large quantities throughout most parts of the plant. In response to herbivore damage, glucosinolate molecules accumulate in their active forms. The presence of glucosinolates influences the resistance of *Brassica rapa* plants to *Delia radicum* insects (Sontowski et al. 2019). Phytoalexins, low‐molecular‐weight compounds with antibacterial properties, accumulate as part of plant defense against insect attacks (Yactayo‐Chang et al. 2020). For example, maize ( *Zea mays* ) affected by the European corn borer ( *Ostrinia nubilalis* ) exhibits high levels of diterpenoid phytoalexins (Huffaker et al. 2011). Plants produce defensins, which are triggered by pathogen attacks and have insecticidal, antibacterial, and antifungal effects (Kovaleva et al. 2020). Defensins inhibit insect digestive enzymes, such as α‐amylase and proteases, serving as a form of defense (Kovaleva et al. 2020).

Nitrogen‐containing compounds are integral to the functioning of plant defense systems. The most important nitrogen‐containing molecules include cyanogenic glycosides, alkaloids, and non‐protein acids. Plants produce alkaloids in response to various stresses. Pyrrolizidine alkaloids (PAs), such as jacobine and erucifoline, are potent compounds that protect plants against insect herbivory (Tlak Gajger and Dar 2021). For example, jacobine significantly contributes to plant protection by greatly increasing thrips mortality (Liu et al. 2017). Cyanogenic glycosides play a crucial role as chemical building blocks in plant defense. The whitefly ( *Bemisia tabaci* ), which feeds on cassava ( *Manihot esculenta* ), activates cyanogenic glycosides, and hydrogen cyanide is converted into beta‐cyanoalanine (Easson et al. 2021). Numerous non‐protein acids, such as gamma‐aminobutyric acid (GABA), beta‐aminobutyric acid, and canavanine, have been studied in relation to plant defense (Huang et al. 2020). Insect feeding, such as that of *Spodoptera littoralis*, promotes the accumulation of GABA (Li, Dou, et al. 2021; Li, Jin, et al. 2021). Canavanine has been shown to repel insects, as certain insect herbivores, such as *Drosophila* species, avoid feeding on plants containing it (Mitri et al. 2009).

## 7 Conclusions and Future Perspectives

This review aims to shed light on the intricate interactions between plants and herbivores, focusing on the key signaling pathways and chemical compounds involved in plant defense mechanisms (Wang et al. 2023). A deeper understanding of these molecular responses enables the identification of potential targets for genetic modification or breeding programs aimed at enhancing plant resistance to herbivory. Such knowledge could lead to the development of crop varieties that are more resilient to insect damage, reducing the dependence on chemical pesticides and promoting more sustainable agricultural practices (War et al. 2012). Additionally, by examining the roles of VOCs and SMs, researchers can uncover potential biocontrol agents that attract natural predators of pests, paving the way for integrating biological control strategies with traditional pest management methods to create more sustainable pest control solutions (Tataridas et al. 2022). Moreover, exploring plant defense mechanisms at the molecular and genetic levels offers significant potential to advance precision agriculture. The creation of genetically modified crops that produce beneficial SMs could provide innovative approaches to reduce insect herbivory, thereby mitigating crop losses and improving food security (Niraula and Fondong 2021). Furthermore, understanding the role of epigenetic regulation in plant defense responses highlights the influence of environmental factors on plant resilience. This knowledge can contribute to the development of sustainable agricultural practices that enhance crop performance under various environmental conditions, ensuring productivity and resilience without compromising yield (Divekar et al. 2022).

The intricate interplay between plants and herbivores, particularly the signaling pathways and chemical compounds involved in plant defense, is a critical area of research that offers promising avenues for sustainable agricultural practices. By enhancing our understanding of these mechanisms, we can develop crops with improved resistance to herbivory, thus reducing the need for chemical pesticides and promoting ecological balance. The potential integration of biocontrol agents and traditional pest management techniques provides an innovative approach to pest control, offering a more environmentally friendly solution to crop protection. Additionally, advances in molecular and genetic research open up new possibilities for precision agriculture, enabling the development of crops that can better withstand insect induced biotic stresses.

## Ethics Statement

The authors have nothing to report.

## Consent

The authors have nothing to report.

## Conflicts of Interest

The authors declare no conflicts of interest.

## Data Availability

Data sharing is not applicable to this article as no datasets were generated or analyzed during the review.
